# Associations of maternal phthalate and bisphenol urine concentrations during pregnancy with childhood blood pressure in a population-based prospective cohort study

**DOI:** 10.1016/j.envint.2020.105677

**Published:** 2020-03-25

**Authors:** Chalana M. Sol, Susana Santos, Alexandros G. Asimakopoulos, Maria-Pilar Martinez-Moral, Liesbeth Duijts, Kurunthachalam Kannan, Leonardo Trasande, Vincent W.V. Jaddoe

**Affiliations:** a The Generation R Study Group, Erasmus MC, University Medical Center Rotterdam, Rotterdam, the Netherlands; b Department of Paediatrics, Erasmus MC – Sophia Children’s Hospital, University Medical Center Rotterdam, Rotterdam, the Netherlands; c Wadsworth Center, New York State Department of Health, and Department of Environmental Health Sciences, School of Public Health, State University of New York at Albany, Albany, NY 12201, USA; d Department of Chemistry, the Norwegian University of Science and Technology (NTNU), 7491 Trondheim, Norway; e Biochemistry Department, Faculty of Science, King Abdulaziz University, Jeddah, Saudi Arabia; f Department of Paediatrics, New York University School of Medicine, New York City, NY 10016, USA; g Department of Environmental Medicine, New York University School of Medicine, New York City, NY 10016, USA; h Department of Population Health, New York University School of Medicine, New York City, NY, USA; i New York University Wagner School of Public Service, New York City, NY 10016, USA; j New York University College of Global Public Health, New York City, NY 10016, USA

**Keywords:** Endocrine disruptor, Phthalate, Bisphenol, Pregnancy, Blood pressure

## Abstract

**Objectives:**

Fetal exposure to phthalates and bisphenols may lead to vascular developmental adaptations, which program later cardiovascular disease. We examined the associations of fetal exposure to phthalates and bisphenols with childhood blood pressure.

**Methods:**

In a population-based, prospective cohort study among 1,064 mother-child pairs, we measured maternal urine phthalate and bisphenol concentrations in first, second and third trimester of pregnancy. We measured childhood blood pressure at the mean age of 9.7 years (standard deviation 0.2 years) old. Analyses were performed for the total group, and for boys and girls separately.

**Results:**

Maternal urine phthalate concentrations were not associated with childhood blood pressure among boys. Higher third trimester maternal urine concentrations of high molecular weight phthalates (HMWP), di-2-ehtylhexylphthalate (DEHP) and di-n-octylphthalate (DNOP) were associated with lower systolic and diastolic blood pressure among girls (p-values < 0.01). Also, higher second trimester maternal urine total bisphenol and bisphenol A concentrations were associated with higher systolic blood pressure among boys (p values < 0.01), but tended to be associated with a lower diastolic blood pressure among girls.

**Conclusions:**

Our results suggest sex-dependent associations of maternal urine phthalate and bisphenol concentrations during pregnancy with childhood blood pressure. Further studies are needed to explore the underlying mechanisms and long term consequences.

## 1. Introduction

Pregnant women are exposed to a variety of endocrine-disrupting chemicals, such as phthalates and bisphenols. (Ye et al., 2008) Phthalates and bisphenols are widely used, including in food packaging, household products and medical devices. (Russo et al., 2019; Schettler, 2006) They are able to pass the placenta and may thereby cause cardiovascular developmental adaptations, which in turn may influence the risk of cardiovascular disease later in life. (Nahar et al., 2015; Mose et al., 2007) It has been hypothesized that exposure to phthalates and bisphenols during fetal life is associated with an increased cardiovascular risk later in life. (Chapalamadugu et al., 2014) The mechanisms by which phthalates and bisphenols affect early-life vascular development may include their influence on estrogen and androgen receptors, thyroid metabolism, the activation of peroxisome proliferator-activated receptors (PPARs) or retinoid X receptors (RXRs), affecting the fetal transcriptome and DNA methylation. (Mattison et al., 2014; Shen et al., 2009; Dolinoy et al., 2007; Solomon et al., 2017; Mou et al., 2018) Due to their influence on estrogen and androgen receptors, the effects of phthalates and bisphenols on cardiovascular health might differ for boys and girls.

The associations of phthalates and bisphenol A (BPA) with cardiovascular risk factors, such as obesity, hypertension and diabetes, have mainly been studied in cross-sectional designs among adult populations and showed inconsistent results. (Ranciere et al., 2015; Trasande et al., 2013; Zhang et al., 2018; Buser et al., 2014; Golestanzadeh et al., 2019) Previous prospective studies on the associations of fetal exposure to phthalates or bisphenols with childhood blood pressure are scarce and also showed inconsistent results. (Vafeiadi et al., 2016; Bae et al., 2017; Vafeiadi et al., 2018; Valvi et al., 2015; Warembourg et al., 2019) Some studies did not show any association of fetal exposure to BPA with childhood blood pressure, while others showed that fetal exposure to BPA was associated with higher diastolic blood pressure. (Vafeiadi et al., 2016; Bae et al., 2017; Warembourg et al., 2019) Studies investigating the associations of fetal phthalate exposure with childhood blood pressure reported sex specific effects. (Vafeiadi et al., 2018; Valvi et al., 2015) The samples from previous studies were small with 230 to 480 mother–child pairs, with follow up until the age of 7 years. A recent study reported associations of prenatal maternal BPA urine concentrations with childhood blood pressure at 6.5–11 years old in 1,277 children form multiple European birth cohorts. (Warembourg et al., 2019) These studies mostly used only one measure of phthalate or bisphenol exposure.

We hypothesized that fetal exposure to higher levels of maternal phthalate and bisphenol concentrations lead to developmental vascular adaptations and subsequent higher blood pressure during childhood. We assessed, in a population-based cohort study from fetal life onwards among 1,064 mother–child pairs, the sex-specific associations of maternal phthalate and bisphenol urine concentrations in first, second and third trimester of pregnancy with childhood blood pressure.

## 2. Materials and methods

### 2.1. Study design

This study was embedded in the Generation R Study, a population-based prospective cohort study from early fetal life onwards in Rotterdam, the Netherlands. (Jaddoe et al., 2012) Phthalate and bisphenol concentrations in urine were measured among a subgroup of 1,405 mothers, whose singleton children also participated in postnatal studies. We excluded mothers without information on phthalate and bisphenol urine concentrations for at least one time point in pregnancy and whose children had no measurement of blood pressure at 10 years. The population for analysis comprised 1,064 mother–child pairs (Supplemental Figure 1). The study has been approved by the Medical Ethical Committee of the Erasmus MC, University Medical Centre in Rotterdam. Written informed consent was obtained from all participants.

### 2.2. Maternal phthalate and bisphenol urine concentrations

Phthalate and bisphenol concentrations were measured in spot urine samples obtained from each woman at three time points during pregnancy (median 12.9 weeks of gestation (25th-75th percentiles 12.1 – 14.5); median 20.4 weeks of gestation (25th-75th percentiles 19.9 – 20.9); median 30.2 weeks of gestation (25th-75th percentiles 29.9 – 30.8)). These periods were considered as first, second and third trimester. Urine samples were collected between February 2004 and July 2005. The analyses of phthalate, bisphenol and creatinine concentrations were performed at the Wadsworth Center, New York State Department of Health, Albany, New York, USA, using previously described methods. (Philips et al., 2018) Urine biomarkers for exposure to phthalate metabolites were grouped according to their molecular weight and parent phthalates into low molecular weight phthalate (LMWP) and high molecular weight phthalate (HMWP), which includes subgroups of di-2-ethylhexylphthalate (DEHP) and di-n-octylphthalate (DNOP) metabolites. Phthalic acid (PA) was analyzed separately as a proxy for total phthalate exposure. Individual phthalates were analyzed as a sensitivity analysis. Bisphenols A (BPA), S (BPS) and F (BPF) were grouped and used as proxy for total bisphenol exposure. Further details on how we grouped the individual phthalates and bisphenols are provided in Supplemental Methods. Weighted molar sums were calculated for the different groups of phthalates and bisphenols. Individual phthalates and bisphenols were included in the groups if < 80% of their concentrations at that time point was below the limit of detection (LOD). All concentrations below the LOD were substituted by LOD divided by the square root of 2 (LOD/√2). (Hornung, 1990) The descriptive statistics of the individual and grouped phthalates and bisphenols investigated are shown for boys and girls in Table 1 (Supplemental Table 1 shows this information for the total group and the limit of detection). The intraclass correlation coefficients between natural log-transformed phthalate and bisphenol urine concentrations across pregnancy were assessed using a single measurement, absolute agreement and two-way mixed effects model and varied between 0.06 and 0.36 (Supplemental Table 1). To account for urine dilution, urine phthalate and bisphenol concentrations were converted to μmol/g creatinine for the metabolite groups. To reduce the potential for exposure misclassification due to temporal variability, we calculated the overall mean exposure during pregnancy by summing the first, second and third trimester phthalate and bisphenol concentrations and dividing by the three time points.

### 2.3. Childhood blood pressure

As described previously, children were invited to visit our research center around the age of 10 years. (Kooijman et al., 2016) We measured systolic and diastolic blood pressure at the right brachial artery four times with one minute intervals, using the validated automatic sphygmomanometer Datascope Accutor Plus (Paramus, NJ, USA) with an appropriate cuff. We calculated the mean value for systolic and diastolic blood pressure using the last three measurements of each participant. (Wong et al., 2006)

### 2.4. Covariates

Information on maternal characteristics, including age, ethnicity, pre-pregnancy body mass index, use of folic acid supplementation, educational level, parity, and maternal smoking habits and alcohol consumption (specifically in first, second and third trimester of pregnancy or during pregnancy) were obtained from questionnaires during pregnancy. Child’s sex was obtained from midwife and hospital records at birth. A Directed Acyclic Graph (DAG) showing the hypothesized relationships between fetal exposure to phthalates and bisphenols, childhood blood pressure and the covariates is presented in Supplemental Figure 2.

### 2.5. Statistical analysis

For all analyses, urine phthalate and bisphenol concentrations were natural log-transformed to reduce variability and account for right skewedness of the distribution and further standardized by the interquartile range (IQR) to ease the interpretation of effect sizes. We constructed standard deviation scores (SDS) [(observed value - mean)/standard deviation (SD)] of the blood pressure based on the sample distribution to enable comparisons of effect sizes. To assess possible bias due to loss-to-follow-up, participants were compared to non-participants using a Chi-squared test, student’s *t*-test or Mann-Whitney *U* test, when applicable. We used linear regression models to assess the associations of maternal urine phthalate and bisphenol concentrations (per time point and overall mean during pregnancy) with childhood blood pressure. Non-linearity of the relationship was visually assessed and ruled out. First, models were adjusted for child’s sex- and age-adjusted height SDS, age and sex only (basic model). Subsequently, potential confounders were identified based on the graphical criteria for confounding by visualizing a DAG. Of these potential confounders, we included those in the models that changed the effect estimates > 10% for at least one of the outcomes. (Santos et al., 2019) To examine the independent associations of maternal first, second and third trimester phthalate and bisphenol concentrations with childhood blood pressure, we created a mutually adjusted model by simultaneously including the exposures at all three time points during pregnancy in the model. Based on our hypothesis of sex-specific effects, we tested for statistical interaction of child’s sex. Since we observed significant interactions for child’s sex, all results were presented for boys and girls separately (Table 3 and Table 4). Previous studies have suggested that the effect of exposure to phthalates and bisphenols on blood pressure could be through DNA methylation changes. (Dolinoy et al., 2007; Solomon et al., 2017; Mou et al., 2018) Because folic acid can influence methylation, the use of folic acid supplementation might modify the associations of phthalates and bisphenols with blood pressure. (Dolinoy et al., 2007; Pauwels et al., 2017) Since we did not observe statistically significant interactions of the interaction (p-values > 0.10), no further stratified analyses were performed. To exclude potential effects of prematurity or low birth weight, we performed a sensitivity analysis excluding children born preterm (meaning being born before 37 weeks of gestation) or with low birth weight (meaning being born below or at the 10th percentile for birth weight as based on gestational age-adjusted SDS for birth weight using North European growth standards as the reference growth curve). (Niklasson et al., 1991) To correct for multiple hypothesis testing, each p-value was compared with a threshold defined as 0.05 divided by the effective number of independent tests estimated based on the correlation structure between the exposures (p-value threshold of 0.0098). (Li et al., 2012) To maintain statistical power and reduce bias related to missing data on covariates, we performed multiple imputation of the covariates according to the Markov Chain Monte Carlo method. The percentage of missing values for covariates ranged from 0 to 20%. Covariates were used as predictor variables and imputed when necessary, blood pressure was used as predictor variable only. Ten imputed datasets were created and no substantial differences were found between the original and imputed datasets. We present results based on pooled imputed datasets. All statistical analyses were performed using the Statistical Package of Social Sciences version 25.0 for Windows (SPSS Inc., Chicago, IL, USA).

## 3. Results

### 3.1. Participant characteristics

Table 2 shows the characteristics for the total group and for boys and girls separately. Non-response analyses showed that non-participating mothers tended to have higher urine phthalate concentrations, were younger, lower educated, more often multiparous, of non-European descent and less likely to take folic acid supplementation or consume alcohol during pregnancy (Supplemental Tables S2 and S3).

### 3.2. Maternal urine phthalate concentrations and childhood blood pressure

Table 3 shows that among boys, maternal urine phthalate concentrations were not associated with childhood systolic or diastolic blood pressure after adjustment for confounders. Among girls, an IQR increase in the natural log-transformed maternal third trimester urine HMWP, DEHP and DNOP concentrations were associated with a 0.14 (95% confidence interval (95% CI) 0.24; 0.05), 0.13 (95% CI 0.22; 0.03) and 0.11 (95% CI 0.21; 0.01) SDS lower systolic blood pressure, and a 0.21 (95% CI 0.30; 0.11), 0.18 (95% CI 0.28; 0.09) and 0.17 (95% CI 0.26; 0.07) SDS lower diastolic blood pressure, respectively (Table 3, basic models given in Supplemental Table S4). Of these associations, only the associations of maternal third trimester urine DNOP concentrations with systolic blood pressure did not remain significant after correction for multiple testing. In the mutually adjusted model, these associations among girls remained significant (Supplemental Table S5). However, the associations of maternal third trimester urine DEHP and DNOP concentrations with offspring systolic blood pressure in the mutually adjusted model did not remain significant after correction for multiple testing. The associations of second trimester maternal urine LMWP concentrations with systolic blood pressure and third trimester maternal urine PA and LMWP concentrations with diastolic blood pressure in girls did not remain significant after adjustment for confounders and correction for multiple testing (Table 3, basic models given in Supplemental Table S4). Higher overall means of maternal urine HMWP, DEHP and DNOP concentrations were also associated with a lower blood pressure among girls, but only the associations of an IQR increase in the natural log transformed overall mean maternal urine HMWP, DEHP and DNOP concentrations with a 0.17 (95% CI 0.26; 0.07), 0.15 (95% CI 0.25; 0.06) and 0.15 (95% CI 0.24; 0.05) SDS lower diastolic blood pressure remained significant after correction for multiple testing (Supplemental Table S6). When examining associations of maternal urine concentrations of individual phthalates with childhood blood pressure, there were multiple associations of higher third trimester maternal urine concentrations of high-molecular weight phthalates such as monobenzylphthalate (mBzBP), mono-(2-ethyl-5-carboxypentyl)phthalate (mECPP), mono-(2-ethyl-5-hydroxyhexyl) phthalate (mEHPP), mono-(2-ethyl-5-oxohexyl)phthalate (mEOHP), mono-[(2-carboxymethyl)hexyl]phthalate (mCMHP) and mono(3-carboxypropyl)phthalate (mCPP) with lower blood pressure among girls only (Supplemental Table S7). When performing a sensitivity analysis concerning children born preterm or at low birth weight, effect estimates of the associations of maternal phthalate urine concentrations with blood pressure during childhood were comparable to the whole group (Supplemental Table S8).

### 3.3. Maternal urine bisphenol concentrations and childhood blood pressure

Table 4 shows that among boys, after adjusting for confounders, an IQR increase in the natural log transformed second trimester maternal urine total bisphenol and BPA concentrations was associated with a 0.13 (95% CI 0.03; 0.23) SDS and 0.14 (95% CI 0.04; 0.23) SDS higher childhood systolic blood pressure. These associations remained significant after correction for multiple testing and also in the mutually adjusted model (Supplemental Table S9). Among girls, higher second trimester maternal urine total bisphenol and BPA concentrations tended to be associated with a lower diastolic blood pressure (Table 4, basic models given in Supplemental Table S10), although this association did not remain after correction for multiple testing. In the mutually adjusted model, an IQR increase in the natural log-transformed urine total bisphenol concentration during second trimester remained significantly associated with a 0.14 (95% CI 0.24; 0.03) SDS lower diastolic blood pressure among girls (Supplemental Table S9). When performing a sensitivity analysis concerning children born preterm or at low birth weight, effect estimates of the associations of maternal bisphenol urine concentrations with blood pressure during childhood were comparable to the whole group (Supplemental Table S11).

## 4. Discussion

### 4.1. Main findings

We observed in a population-based prospective cohort study that the associations of exposure to phthalates and bisphenols during pregnancy with blood pressure at 10 years old differ between boys and girls. Higher fetal exposure to phthalates is associated with lower blood pressure among girls, while higher fetal exposure to bisphenols is associated with higher blood pressure among boys at age 10 years.

### 4.2. Interpretation of main findings

Because of the wide-spread use of phthalates and bisphenols and their ability to cross the placenta, exposure to these potential harmful metabolites probably starts before birth. Exposure to phthalates and bisphenols during fetal life may lead to vascular developmental adaptations, with subsequently an increase in the risk of hypertension and cardiovascular disease in later life.

Few studies assessed the associations of fetal exposure to phthalates with childhood blood pressure. (Vafeiadi et al., 2018; Valvi et al., 2015; Warembourg et al., 2019) In a study among 350 Spanish mother–child pairs, higher maternal urine total HMWP, total LMWP and monoethylphthalate (mEP) concentrations were associated with lower systolic blood pressure among girls at 4 and 7 years old. (Valvi et al., 2015) No association was observed for diastolic blood pressure among girls and boys. In a Greek study among 230 mother–child pairs, higher maternal urine mono-isobutylphthalate (mIBP) concentrations were associated with lower diastolic blood pressure at 4–6 years old, especially among boys. (Vafeiadi et al., 2018) Higher maternal urine DEHP concentrations tended to be associated with lower systolic and diastolic blood pressure. Results from a multi-pollutant approach study among 1,277 children from different birth cohorts in Europe suggested that maternal urine phthalate concentrations were not associated with childhood blood pressure around 8 years of age (range 6.5–11 years). (Warembourg et al., 2019) However, this study also reported that higher childhood urine monobenzylphthalate (mBzBP) concentrations were associated with lower systolic blood pressure during childhood. (Warembourg et al., 2019) In the present study, higher fetal exposure to (high molecular weight) phthalates was associated with lower blood pressure at 10 years old among girls. Third trimester maternal mBzBP was also associated with lower systolic and diastolic blood pressure among girls, which is in line with the previous study. The lowering effect on blood pressure by third trimester phthalates observed in our study is in line with the few previous longitudinal studies in humans. However, it is in contrast to our original hypothesis, which was mainly based on animal studies and cross-sectional studies in humans. (Lu et al., 2018) It is possible that these studies have been hindered by residual confounding, based on the possibility of unhealthy life choices leading to both increased phthalate exposure and cardiovascular risk. The prospective association of fetal exposure to phthalates with lower blood pressure in our study seems to be restricted to girls, which is in line with the study from the INMA-Sabadell Birth Cohort Study, but not with the results from the Rhea Study. The exposure to phthalates was assessed during first trimester in the Rhea Study, while in the INMA-Sabadell Birth Cohort Study the averages of first and third trimester exposures were used, which is more in line with our study. Also, previous studies might have been underpowered to assess sex-specific effects, which might explain the different findings across studies. Altogether, results from our and other prospective studies suggest that higher fetal exposure to phthalates may be associated with lower blood pressure among girls only.

Previous studies examining the associations of maternal bisphenol concentrations with childhood blood pressure have only focused on BPA. No association between maternal urine BPA concentrations and childhood blood pressure was found in 235 Greek mothers and their 4-year-old children. (Vafeiadi et al., 2016) In another study among 486 mother–child pairs, maternal mid-pregnancy urine BPA concentration was positively associated with diastolic blood pressure at 4 years old among boys and with systolic blood pressure at 4 years old among girls. (Bae et al., 2017) In the most recent study including 1,277 children around 8 years old from Europe, higher maternal urine BPA concentrations during pregnancy were also associated with higher diastolic blood pressure. (Warembourg et al., 2019) In the present study, higher fetal exposure to bisphenols was associated with higher blood pressure at 10 years old among boys, but not among girls. The findings from this study are not fully in line with previous studies in smaller samples with 4-year-old children, which might be due to different ages at which blood pressure was measured. It is also possible that power issues may have led to the absence of an association in the previous Greek study. Thus, so far results from studies assessing the associations of fetal exposure to bisphenol concentrations with blood pressure are not consistent. However, findings from the largest study suggested an association of higher maternal urine BPA concentrations with an increase in diastolic blood pressure during childhood, which is in line with our findings.

Our results suggest that exposure to endocrine disruptors during fetal life may have long-lasting effects on blood pressure during childhood. Due to the observational design of this study, we cannot draw conclusions about causality. The observed effect estimates were small but are of interest from a developmental perspective since blood pressure tends to track into adulthood and even subclinical differences during childhood are related to the development of cardiovascular disease in later life. (Chen and Wang, 2008)

### 4.3. Possible underlying mechanisms

Numerous underlying mechanisms have been proposed for the hypothesized adverse effects of exposure to phthalates and bisphenols on blood pressure. It has been suggested that exposure to phthalates causes an adverse inflammatory environment through oxidative stress. (Ferguson et al., 2014) This hypothesis of an adverse inflammatory environment is not in line with our results, which actually show a lowering effect of phthalates on blood pressure. It has previously been shown that exposure to DEHP during fetal life lowered blood pressure during adulthood among rats. (Martinez-Arguelles et al., 2013) The suggested mechanism behind this is that DEHP causes reduced production of aldosterone in the adrenal gland because of a decrease in expression of the angiotensin II receptor, possibly by an epigenetic mechanism. (Martinez-Arguelles et al., 2014; Martinez-Arguelles et al., 2011) For BPA, it has been proposed that BPA damages kidney cells, possibly via inducing oxidative stress. (Bosch-Panadero et al., 2018; Nunez et al., 2018) The mechanisms for the sex-specific effects are not completely known, but could involve sex-specific differences in PPAR-activity of phthalates and the estrogenic effect of BPA. (Jalouli et al., 2003; Huang and Chen, 2017; Mendelsohn and Karas, 2005)

### 4.4. Methodological considerations

An important strength of this study is the population-based cohort design from fetal life onwards, with repeated measurements of multiple maternal phthalate and bisphenol concentrations and measures of childhood blood pressure in a large number of mother–child pairs. Selection bias due to selective loss to follow-up would be of concern in any study if the associations of prenatal phthalate and bisphenol concentrations with childhood blood pressure are different between participants and non-participants. This seems unlikely, but cannot be excluded with certainty. Furthermore, we measured maternal urine phthalate and bisphenol concentrations at one moment per trimester. It is possible that one urine sample per trimester does not accurately represent the concentration during the whole trimester because of the reported short biological half-lives of phthalates and bisphenols. (Mattison et al., 2014; Braun et al., 2013) However, it has been found that a single urine sample reflects phthalate exposure up to three months. (Hauser et al., 2004) Variability has also been reported to be biomarker specific, with strong correlations for LWMP metabolites and reasonable correlations for BPA and DEHP metabolites. (Braun et al., 2012; Mahalingaiah et al., 2008) In our study, we observed high variability for bisphenols and moderate variability for phthalates across pregnancy. We replaced the values of phthalate and bisphenol urine concentrations below LOD by LOD divided by the square root of 2. Single substitution is acceptable when the proportion of values below the LOD is low but might introduce bias in the results as the proportion of values below LOD increases. Overall, the detection rates of the compounds are reasonable (< 35% below LOD), except for monocyclohexyl-phthalate (mCHP) in the first trimester (79.5% of values < LOD), BPS in the second trimester (70.5% of values < LOD) and BPF in the first and third trimester (59.6 respectively 71.5% values < LOD). We cannot disregard the possibility that, for these specific compounds, we did not have enough variability to detect associations. Finally, we collected information on many potential confounders. However, as in any observational study, residual confounding due to unmeasured lifestyle characteristics might remain an issue. Unfortunately, we did not have information on phthalate or bisphenol urine concentrations during childhood available in the full group. This might be a limitation, since these concentrations might be related to both the pregnancy concentrations and the outcomes of interest. Previous studies reported associations of childhood phthalate or bisphenol urine concentrations with their blood pressure. (Trasande et al., 2013; Khalil et al., 2014) However, these studies might suffer from reverse causality due to their cross-sectional design. Further studies are needed to take childhood concentrations into account.

## 5. Conclusions

In this population-based prospective cohort study, we found sex-specific differences in the association of maternal urine phthalate and bisphenol concentrations during pregnancy with childhood blood pressure at 10 years old. Higher fetal exposure to (high molecular weight) phthalates is associated with lower blood pressure among girls and higher fetal exposure to bisphenols is associated with higher blood pressure among boys. These results should be considered as hypothesis generating and further studies are needed for replication and identification of underlying mechanisms.

## Supplementary Material

SUpplementary material

## Figures and Tables

**Table 1:** Urine concentrations of phthalates and bisphenols during pregnancy, stratified in boys and girls.

	First trimester Median (25th-75th percentile)		Second trimester Median (25th-75th percentile)		Third trimester Median (25th-75th percentile)	
	Boys n = 538	Girls n = 526	Boys n = 538	Girls n = 526	Boys n = 538	Girls n = 526
**Phthalic Acid (PA) (nmol/L)**	344.7 (180.7 – 681.4)	335.2 (178.7 – 742.1)	935.0 (376.7 – 1715.6)	857.8 (337.7 – 1621.9)	387.8 (190.7 – 800.3)	420.3 (218.1 – 779.3)
**Low-molecular-weight phthalates (LMWP) (nmol/L)**	1092.3 (474.6 – 2751.4)	1093.5 (403.8 – 3069.0)	527.4 (238.9 – 1281.0)	555.5 (215.1 – 1610.8)	964.2 (364.2 – 2349.2)	1014.1 (410.6 – 2671.0)
Monomethylphthalate (mMP) (nmol/L)	28.5 (15.0 – 54.5)	31.7 (15.6 – 55.8)	19.0 (10.4 – 36.3)	18.8 (9.3 – 34.5)	20.3 (10.4 – 39.6)	23.1 (11.4 – 47.7)
Monoethylphthalate (mEP) (nmol/L)	703.2 (210.3 – 2388.4)	726.2 (207.1 – 2524.4)	333.4 (112.9 – 970.4)	346.2 (120.2 – 1332.6)	593.9 (209.4 – 1962.3)	689.1 (233.0 – 2205.8)
Mono-isobutylphthalate (mIBP) (nmol/L)	98.6 (45.9 – 205.7)	92.9 (40.2 – 194.3)	39.7 (20.1 – 78.7)	39.4 (18.9 – 76.2)	69.9 (38.0 – 141.0)	80.5 (41.4 – 157.2)
Mono-n-butylphthalate (mBP) (nmol/L)	75.7 (31.3 – 139.9)	70.8 (30.3 – 137.5)	43.1 (25.2 – 81.7)	41.1 (21.4 – 77.3)	53.2 (27.2 – 100.6)	53.2 (27.8 – 113.3)53.
**High-molecular-weight phthalates (HMWP) (nmol/L)**	216.1 (112.6 – 420.8)	209.5 (115.8 – 376.8)	130.2 (75.2 – 242.5)	126.3 (67.9 – 224.0)	160.2 (92.8 – 270.0)	169.8 (98.6 – 316.4)
Monobenzylphthalate (mBzBP) (nmol/L)	21.2 (8.5 – 47.9)	22.4 (9.2 – 46.0)	21.0 (8.1 – 39.0)	19.3 (8.5 – 41.3)	11.5 (4.2 – 24.0)	13.3 (4.1 – 25.1)
Mono-hexylphthalate (mHxP) (nmol/L)	0.9 (0.3 – 2.1)	0.9 (0.3 – 1.8)	NA	NA	NA	NA
Mono-2-heptylphthalate (mHpP) (nmol/L)	2.1 (< LOD – 5.4)	2.0 (< LOD – 5.3)	NA	NA	NA	NA
Monocyclohexyl-phthalate (mCHP) (nmol/L)	< LOD (< LOD – < LOD)	< LOD (< LOD – < LOD)	NA	NA	NA	NA
***Di-2-ehtylhexylphthalate (DEHP)* (nmol/L)**	174.2 (90.0 – 328.6)	168.4 (88.8 – 298.6)	96.9 (54.1 – 184.2)	93.9 (48.7 – 174.3)	134.6 (74.7 – 227.5)	143.1 (81.4 – 272.8)
Mono-(2-ethyl-5-carboxy-pentyl)phthalate (mECPP) (nmol/L)	52.2 (25.9 – 101.0)	51.7 (26.4 – 97.8)	34.8 (17.9 – 63.7)	31.5 (17.8 – 59.1)	54.4 (29.3 – 104.8)	59.6 (32.7 – 114.5)
Mono-(2-ethyl-5-hydroxy-hexyl)phthalate (mEHHP) (nmol/L)	41.7 (20.7 – 81.4)	38.9 (19.5 – 70.0)	19.3 (10.6 – 37.1)	17.5 (9.5 – 34.5)	32.8 (16.8 – 62.2)	38.7 (18.5 – 73.7)
Mono-(2-ethyl-5-oxohexyl)phthalate (mEOHP) (nmol/L)	26.9 (12.1 – 55.7)	26.0 (12.1 – 48.8)	25.8 (12.4 – 56.7)	22.6 (11.5 – 51.8)	23.4 (12.7 – 44.1)	25.0 (13.9 – 50.3)
Mono-[(2-carboxymethyl)-hexyl] phthalate (mCMHP) (nmol/L)	44.1 (24.5 – 83.4)	45.4 (24.4 – 81.1)	12.5 (7.1 – 23.4)	13.0 (6.9 – 22.9)	9.9 (5.6 – 20.0)	11.8 (6.1 – 21.4)
***Di-n-octylphthalate (DNOP)***	5.6 (3.1 – 11.0)	5.7 (2.9 – 10.4)	3.5 (2.1 – 6.8)	3.4 (1.9 – 6.5)	7.0 (3.7 – 12.2)	7.2 (3.9 – 12.6)
Mono(3-carboxypropyl)-phthalate (mCPP) (nmol/L)	5.6 (3.1 – 11.0)	5.7 (2.9 – 10.4)	3.5 (2.1 – 6.8)	3.4 (1.9 – 6.5)	7.0 (3.7 – 12.2)	7.2 (3.9 – 12.6)
**Bisphenols (nmol/L)**	9.8 (3.5 – 21.6)	8.7 (3.6 – 20.0)	6.4 (3.2 – 13.4)	6.1 (2.9 – 13.7)	8.4 (4.1 – 17.6)	10.0 (4.6 – 19.8)
Bisphenol A (BPA) (nmol/L)	5.2 (1.2 – 12.4)	4.5 (1.0 – 13.2)	5.9 (2.7 – 12.6)	5.5 (2.4 – 12.0)	6.0 (2.6 – 11.9)	7.2 (2.9 – 14.3)
Bisphenol S (BPS) (nmol/L)	0.7 (< LOD – 2.6)	0.6 (< LOD – 2.3)	< LOD (< LOD – 0.5)	< LOD (< LOD – 0.4)	NA	NA
Bisphenol F (BPF) (nmol/L)	< LOD (< LOD – 1.8)	< LOD (< LOD – 2.4)	NA	NA	< LOD (< LOD – 2.9)	< LOD (< LOD – 1.4)

Values represent medians (25th-75th percentiles) of absolute urine concentrations of grouped exposures (in nmol/L urine) and individual exposures (in nmol/L urine) with concentrations below the limit of detection imputed as limit of detection/square root of 2. Only individual exposures with < 80% of values below LOD for at least one of the trimesters are included in the calculation of the grouped exposures and are thus included in this table.

NA: not applicable due to >80% of concentrations below limit of detection.

**Table 2:** Characteristics of mothers and their children.

	Total group n = 1,064	Boys n = 538 (50.6%)	Girls n = 526 (49.4)
**Maternal characteristics**			
Age at enrolment, mean (SD) (years)	30.9 (4.6)	30.9 (4.6)	30.8 (4.6)
Parity, n (%)			
Nullipara	666 (62.9%)	338 (63.1%)	328 (62.8%)
Multipara Ethnicity, n (%)	392 (37.1%)	198 (36.9%)	194 (37.2%)
European	692 (65.4%)	356 (66.5%)	336 (64.2%)
Non-European Education, n (%)	366 (34.6%)	179 (33.5%)	187 (35.8%)
Low	67 (6.5%)	32 (6.2%)	35 (6.9%)
Middle	470 (39.5%)	199 (38.3%)	208 (40.6%)
High	556 (54.0%)	289 (55.6%)	276 (52.4%)
Pre-pregnancy BMI, median (95% range) (kg/m2)	22.7 (18.5 – 34.9)	22.7 (18.6 – 35.0)	22.7 (18.4 – 35.0)
Folic acid supplementation, n (%), yes	708 (83.2%)	364 (83.7%)	344 (82.7%)
Smoking during pregnancy, n (%), yes	222 (22.9%)	110 (22.9%)	112 (22.9%)
First trimester, n (%), yes	189 (20.0%)	94 (20.0%)	95 (19.9%)
Second trimester, n (%), yes	96 (10.3%)	47 (9.8%)	49 (10.8%)
Third trimester, n (%), yes	97 (10.5%)	54 (11.3%)	43 (9.6%)
Alcohol consumption during pregnancy (any), n (%), yes	581 (60.4%)	305 (63.5%)	276 (57.3%)
First trimester, n (%), yes	492 (52.1%)	263 (55.7%)	229 (48.4%)
Second trimester, n (%), yes	342 (36.7%)	184 (38.8%)	158 (34.5%)
Third trimester, n (%), yes	361 (39.1%)	199 (42.0%)	162 (30.8%)
**Child characteristics**			
Age, mean (SD) (years)	9.7 (0.2)	9.7 (0.3)	9.7 (0.2)
BMI, median (95% range) (kg/m^2^)	16.8 (14.0 – 24.9)	17.3 (14.0 – 24.5)	16.9 (14.0 – 25.2)
Average diastolic blood pressure, mean (SD) (mmHg)^a^	58.6 (6.7)	57.9 (6.8)	59.3 (6.6)
Average systolic blood pressure, mean (SD) (mmHg)^a^	103.0 (8.2)	102.5 (7.9)	103.6 (8.4)

Values represent mean (SD), median (95% range) or number of subjects (valid %).

a The average of the last 3 of 4 measurements was used.

**Table 3:** Associations of maternal phthalate urine concentration during pregnancy with childhood blood pressure at 10 years, stratified for boys and girls.

		Measures of blood pressure at 10 years (in standard deviation scores, 95% confidence interval)					
		Mean systolic blood pressure			Mean diastolic blood pressure		
Exposure	Trimester	Boys	Girls	p-value^$^	Boys	Girls	p-value^$^
PA	First trimester	0.04 (−0.05; 0.14)	−0.03 (−0.13; 0.07)	0.322	0.01 (−0.09; 0.11)	−0.03 (−0.13; 0.07)	0.311
	Second trimester	0.07 (−0.04; 0.18)	0.02 (−0.10; 0.14)	0.626	0.02 (−0.10; 0.14)	0.01 (−0.11; 0.14)	0.777
	Third trimester	0.03 (−0.07; 0.13)	−0.03 (−0.15; 0.08)	0.412	−0.05 (− 0.16; 0.06)	−0.12 (−0.24; −0.01)*	0.254
LMWP	First trimester	0.04 (−0.07; 0.14)	0.04 (−0.08; 0.15)	0.973	0.04 (−0.07; 0.16)	0.01 (−0.11; 0.12)	0.363
	Second trimester	0.05 (−0.06; 0.16)	0.11 (−0.01; 0.23)	0.457	−0.06 (−0.18; 0.06)	0.09 (−0.03; 0.21)	0.203
	Third trimester	−0.00 (−0.12; 0.11)	−0.03 (−0.15; 0.10)	0.797	−0.04 (−0.17; 0.08)	−0.12 (−0.25; 0.01)	0.218
HMWP	First trimester	0.03 (−0.07; 0.12)	−0.04 (−0.15; 0.06)	0.346	0.06 (−0.04; 0.15)	−0.10 (−0.20; 0.01)	0.025^#^
	Second trimester	0.05 (−0.05; 0.14)	−0.04 (−0.14; 0.06)	0.234	0.05 (−0.05; 0.15)	−0.10 (−0.20; −0.00)*	0.037^#^
	Third trimester	−0.04 (−0.13; 0.06)	−0.14 (−0.24; 0.05)^†^	0.081^#^	−0.03 (−0.14; 0.08)	−0.21 (−0.30; −0.11)^†^	0.014^#^
DEHP	First trimester	0.03 (−0.07; 0.12)	−0.04 (−0.14; 0.07)	0.362	0.05 (−0.05; 0.15)	−0.09 (−0.19; 0.02)	0.047^#^
	Second trimester	0.04 (−0.05; 0.14)	−0.05 (−0.14; 0.05)	0.218	0.06 (−0.04; 0.16)	−0.09 (−0.19; 0.06)	0.034^#^
	Third trimester	−0.04 (−0.14; 0.06)	−0.13 (−0.22; −0.03)^†^	0.136	−0.04 (−0.14; 0.07)	−0.18 (−0.28; −0.09)^†^	0.033^#^
DNOP	First trimester	0.02 (−0.07; 0.10)	−0.04 (−0.14; 0.06)	0.390	0.02 (−0.07; 0.11)	−0.07 (−0.17; 0.03)	0.204
	Second trimester	0.04 (−0.05; 0.13)	−0.06 (−0.16; 0.05)	0.219	−0.01 (−0.11; 0.09)	−0.04 (−0.15; 0.07)	0.695
	Third trimester	0.03 (−0.06; 0.13)	−0.11 (−0.21; −0.01)*	0.038^#^	−0.01 (−0.11; 0.09)	−0.17 (−0.26; −0.07)^†^	0.047^#^

Values are regression coefficients (95% confidence interval) from linear regression models that reflect the difference in blood pressure in SDS for an interquartile range increase in each natural log-transformed phthalate or bisphenol (in μmol/g creatinine) in boys or girls. Model includes child’s age and standardized height and maternal age, education, parity, ethnicity, pre-pregnancy body mass index, alcohol consumption and smoking habits (specifically in early, mid and late pregnancy).

$ Values are p-values from associations from linear regression models that reflect the significance of the interaction of gender and exposure on childhood blood pressure.

DEHP, di-2-ethylhexylphthalate; DNOP, di-n-octylphthalate; HMWP, high molecular weight phthalates; LMWP, low molecular weight phthalate; PA, phthalic acid.

# p-value for interaction term < 0.10.

* p-value < 0.05.

† Significant after correction for multiple testing (p-value threshold of 0.0098).

**Table 4:** Associations of maternal bisphenol urine concentration during pregnancy with childhood blood pressure at 10 years, stratified for boys and girls.

		Measures of blood pressure at 10 years (in standard deviation scores, 95% confidence interval)					
		Mean systolic blood pressure			Mean diastolic blood pressure		
Exposure	Trimester	Boys	Girls	p-value^$^	Boys	Girls	p-value^$^
BP	First trimester	0.04 (−0.07; 0.14)	−0.05 (−0.16; 0.05)	0.266	0.08 (−0.04; 0.19)	0.01 (−0.10; 0.12)	0.382
	Second trimester	0.13 (0.03; 0.23)^†^	−0.08 (−0.18; 0.02)	0.004^#^	0.04 (−0.06; 0.15)	−0.12 (−0.23; −0.02)*	0.038^#^
	Third trimester	0.01 (−0.09; 0.11)	0.02 (−0.09; 0.13)	0.893	0.01 (−0.10; 0.12)	−0.06 (−0.17; 0.05)	0.391
BPA	First trimester	0.04 (−0.07; 0.15)	−0.07 (−0.18; 0.03)	0.140	0.04 (−0.08; 0.16)	0.01 (−0.10; 0.11)	0.570
	Second trimester	0.14 (0.04; 0.23)^†^	−0.08 (−0.18; 0.02)	0.002^#^	0.05 (−0.05; 0.15)	−0.12 (−0.22; −0.02)*	0.032^#^
	Third trimester	−0.01 (−0.10; 0.09)	0.05 (−0.05; 0.15)	0.369	0.02 (−0.08; 0.12)	−0.02 (−0.12; 0.08)	0.552
BPS	First trimester	0.06 (−0.05; 0.18)	−0.02 (−0.14; 0.10)	0.373	0.11 (−0.02; 0.23)	0.03 (−0.09; 0.15)	0.441
	Second trimester	−0.05 (−0.15; 0.05)	0.04 (−0.06; 0.14)	0.173	−0.04 (−0.14; 0.07)	0.01 (−0.10; 0.11)	0.463
	Third trimester	NA	NA	NA	NA	NA	NA
BPF	First trimester	0.05 (−0.07; 0.16)	−0.05 (−0.16; 0.07)	0.256	0.08 (−0.03; 0.20)	−0.03 (−0.14; 0.11)	0.142
	Second trimester	NA	NA	NA	NA	NA	NA
	Third trimester	0.02 (−0.09; 0.12)	−0.09 (−0.21; 0.02)	0.127	−0.05 (−0.16; 0.07)	−0.12 (−0.23; 0.00)	0.584

Values are regression coefficients (95% confidence interval) from linear regression models that reflect the difference in blood pressure in SDS for an interquartile range increase in each natural log-transformed bisphenol (in μmol/g creatinine) in boys or girls. Model includes child’s age and standardized height and maternal age, education, parity, ethnicity, pre-pregnancy body mass index, alcohol consumption and smoking habits (specifically in early, mid and late pregnancy).

$ Values are p-values from associations from linear regression models that reflect the significance of the interaction of gender and exposure on childhood blood pressure. BP, bisphenols; BPA, bisphenol A; BPF, bisphenol F; BPS, bisphenol S; NA: not applicable due to > 80% of concentrations below limit of detection.

# p-value for interaction term < 0.10.

* p-value < 0.05.

† Significant after correction for multiple testing (p-value threshold of 0.0098).

## Abbreviations:

PPARs: peroxisome proliferator-activated receptors
RXRs: retinoid X receptors
BPA: bisphenol A
LMWP: low molecular weight phthalate
HMWP: high molecular weight phthalate
DEHP: di-2-ethylhexylphthalate
DNOP: di-n-octylphthalate
PA: phthalic acid
BPS: bisphenol S
BPF: bisphenol F
LOD: limit of detection
DAG: Directed Acyclic Graph
IQR: interquartile range
SDS: standard deviation scores
SD: standard deviation
95% CI: 95% confidence interval
mBzBP: monobenzylphthalate
mECPP: mono-(2-ethyl-5-carboxypentyl)phthalate
mEHPP: mono-(2-ethyl-5-hydroxyhexyl)phthalate
mEOHP: mono-(2-ethyl-5-oxohexyl) phthalate
mCMHP: mono-[(2-carboxymethyl)hexyl]phthalate
mCPP: mono(3-carboxypropyl)phthalate
mEP: monoethylphthalate
mIBP: mono-isobutylphthalate
