# Supplementary material for: Associations of maternal phthalate and bisphenol urine concentrations during pregnancy with childhood blood pressure in a population-based prospective cohort study
Source: Environ Int. Author manuscript; Available in PMC 2020 Jul 12. (PMC7354351; doi:10.1016/j.envint.2020.105677)
Supplement: SUpplementary material [file NIHMS1606061-supplement-SUpplementary_material.docx]

**Online Supporting Material**

**Associations of maternal phthalate and bisphenol urine concentrations during pregnancy with childhood blood pressure in a population-based prospective cohort study.**

***Short title: Phthalates and bisphenols and childhood blood pressure***

Chalana M. Sol MD, Susana Santos PhD, Alexandros G. Asimakopoulos PhD, Maria-Pilar Martinez-Moral PhD, Liesbeth Duijts MD PhD, Kurunthachalam Kannan PhD, Leonardo Trasande MD MPP, Vincent WV Jaddoe MD PhD

**Corresponding author:** Vincent WV Jaddoe, The Generation R Study Group (Na 29-08), Erasmus MC, University Medical Center Rotterdam, PO Box 2040, 3000 CA, Rotterdam, The Netherlands. Phone: +31 10 7043405; e-mail address: [v.jaddoe@erasmusmc.nl](mailto:v.jaddoe@erasmusmc.nl).

**CONTENTS**

**Supplemental Figure 1.** Flowchart of participants included in the study.

**Supplemental Methods.**

**Supplemental Table S1.** Urine concentrations of phthalates and bisphenols during pregnancy, total group (n = 1,064).

**Supplemental Figure 2.** Directed Acyclic Graph showing the hypothesized relationship between fetal exposure to phthalates and bisphenols, childhood blood pressure and the covariates.

**Supplemental Table S2.** Urine concentrations of phthalates and bisphenols during pregnancy in participants and non-participants (n = 315).

**Supplemental Table S3.** Comparison between participants and non-participants.

**Supplemental Table S4.** Associations of maternal urine phthalate concentration during pregnancy with childhood blood pressure at 10 years, basic model, stratified for boys and girls.

**Supplemental Table S5.** Associations of maternal urine phthalate concentration during pregnancy with childhood blood pressure at 10 years, mutually adjusted model, stratified for boys and girls.

**Supplemental Table S6.** Associations of maternal urine phthalate and bisphenol concentration during pregnancy with childhood blood pressure at 10 years, stratified for boys and girls, overall mean.

**Supplemental Table S7.** Associations of maternal urine individual phthalate concentration during pregnancy with childhood blood pressure at 10 years, stratified for boys and girls.

**Supplemental Table S8.** Associations of maternal urine phthalate concentration during pregnancy with childhood blood pressure at 10 years in children born at term not at low birth weight, stratified for boys and girls (n = 944).

**Supplemental Table S9.** Associations of maternal urine bisphenol concentration during pregnancy with childhood blood pressure at 10 years, mutually adjusted model, stratified for boys and girls.

**Supplemental Table S10.** Associations of maternal urine bisphenol concentration during pregnancy with childhood blood pressure at 10 years, basic model, stratified for boys and girls.

**Supplemental Table S11.** Associations of maternal urine bisphenol concentration during pregnancy with childhood blood pressure at 10 years in children born at term not at low birth weight, stratified for boys and girls (n = 944).

**Supplemental Figure 1.** Flowchart of participants included in the study.

| Mothers with information on exposure to phthalates and bisphenols during pregnancy with singleton live-born children  1,405 | |  |  |
| --- | --- | --- | --- |
|  |  |  | Excluded: Mothers without information on exposure to phthalates and bisphenols for at least one time point in pregnancy  n = 26  First trimester: n = 9  Second trimester: n = 14  Third trimester: n = 13 |
|  |  |  |  |
| Mothers with information on exposure to phthalates and bisphenols at all three time points in pregnancy with singleton live-born children  n = 1,379 | |  |  |
|  |  |  | Excluded: Children without any measurement of blood pressure at 10 years  n = 315 |
|  |  |  |  |
| Mothers with information on exposure to phthalates and bisphenols at all three time points in pregnancy and their singleton live-born children with a measurement of blood pressure  n = 1,064 | |  |  |

This is a 2-column fitting image.

**Supplemental Methods**

The low-molecular weight phthalate (LMWP) group in all trimesters consists of monomethylphthalate (mMP), monoethylphthalate (mEP), mono-isobutylphthalate (mIBP) and mono-n-butylphthalate (mBP). The high-molecular-weight phthalate (HMWP) group in the first trimester consists of monobenzylphthalate (mBzBP), mono-hexylphthalate (mHxP), mono-2-heptylphthalate (mHpP), monocyclohexyl-phthalate (mCHP) and the di-2-ethylhexylphtalate (DEHP) and di-n-octylphthalate (DNOP) groups. The high-molecular-weight phthalate group in the second and third trimester consists of monobenzylphthalate (mBzBP) and the di-2-ethylhexylphthalate (DEHP) and di-n-octylphthalate groups (DNOP). The di-2-ethylhexylphthalate (DEHP) group in all trimesters consists of mono-(2-ethyl-5-carboxypentyl)phthalate (mECPP), mono-(2-ethyl-5-hydroxyhexyl)phthalate (mEHHP), mono-(2-ethyl-5-oxohexyl)phthalate (mEOHP) and mono[(2-carboxymethyl)-hexyl]phthalate (mCMHP). The di-n-octylphthalate (DNOP) group in all trimesters consists of mono(3-carboxypropyl)-phthalate (mCPP). The bisphenol group consists of bisphenol A (BPA), bisphenol S (BPS) and bisphenol F (BPF) in the first trimester, bisphenol A (BPA) and bisphenol S (BPS) in the second trimester and bisphenol A (BPA) and bisphenol F (BPF) in the third trimester.

**Supplemental Table 1.** Urine concentrations of phthalates and bisphenols during pregnancy, total group (n = 1,064).

|  | Limit of detection (nmol/L) | **First trimester**  Median (25^th^-75^th^ percentile) | **Percentage <LOD**  (%) | **Second trimester**  Median (25^th^-75^th^ percentile) | **Percentage <LOD** | **Third trimester**  Median (25^th^-75^th^ percentile) | **Percentage <LOD** | **ICC** |
| --- | --- | --- | --- | --- | --- | --- | --- | --- |
| **Phthalic Acid (PA)** (nmol/L) | 6.68 | 343.1  (180.3 – 724.8) | 0.4 | 878.5  (349.1 – 1662.6) | 0.1 | 402.2  (200.4 – 792.3) | 0.5 | 0.20 |
| **Low-molecular-weight phthalates (LMWP)**  (nmol/L) | - | 1092.6  (429.2 – 2903.0) | - | 533.6  (224.3 – 1422.3) | - | 982.0  (388.4 – 2480.7) | - | 0.36 |
| Monomethylphthalate (mMP) (nmol/L) | 0.33 | 29.8  (15.1 – 54.9) | 0.1 | 18.9  (9.8 – 34.9) | 0.1 | 21.6  (10.9 – 44.5) | 0.6 |  |
| Monoethylphthalate (mEP) (nmol/L) | 0.31 | 713.7  (208.1 – 2437.7) | 0.1 | 341.3  (118.7 – 1090.3) | 0 | 642.7  (217.5 – 2055.0) | 0 |  |
| Mono-isobutylphthalate (mIBP) (nmol/L) | 0.40 | 95.9  (42.7 – 203.0) | 0.2 | 39.5  (19.6 – 77.5) | 0 | 75.2  (40.3 – 151.9) | 0.4 |  |
| Mono-n-butylphthalate (mBP) (nmol/L) | 0.63 | 72.8  (30.6 – 138.7) | 0.7 | 41.8  (23.3 – 81.3) | 0 | 53.1  (27.4 – 107.1) | 0.2 |  |
| **High-molecular-weight phthalates (HMWP)** (nmol/L) | **-** | 214.3  (114.6 – 394.2) | - | 128.0  (70.4 – 236.5) | - | 165.9  (95.5 – 292.9) | - | 0.19 |
| Monobenzylphthalate (mBzBP) (nmol/L) | 0.59 | 22.1  (8.8 – 47.2) | 8.6 | 19.9  (8.3 – 40.5) | 1.6 | 12.0  (4.1 – 24.5) | 3.8 |  |
| Mono-hexylphthalate (mHxP) (nmol/L) | 0.24 | 0.9  (0.3 – 1.9) | 23.3 | NA | 99.0 | NA | 98.0 |  |
| Mono-2-heptylphthalate (mHpP) (nmol/L) | 0.97 | 2.1  (<LOD – 5.3) | 35.2 | NA | 96.8 | NA | 98.4 |  |
| Monocyclohexyl-phthalate (mCHP) (nmol/L) | 0.16 | <LOD  (<LOD – <LOD) | 79.5 | NA | 99.2 | NA | 94.8 |  |
| ***Di-2-ehtylhexylphthalate (DEHP)*** (nmol/L) | **-** | 171.4  (89.2 – 313.5) | - | 95.4  (50.9 – 178.3) | - | 138.6  (77.5 – 250.2) | - | 0.17 |
| Mono-(2-ethyl-5-carboxy-pentyl)phthalate (mECPP) (nmol/L) | 0.94 | 52.1  (26.1 – 99.6) | 0.1 | 33.1  (17.9 – 61.9) | 0.1 | 57.9  (30.7 – 109.2) | 0 |  |
| Mono-(2-ethyl-5-hydroxy-hexyl)phthalate (mEHHP) (nmol/L) | 0.27 | 40.3  (20.1 – 77.0) | 0.2 | 18.2  (10.0 – 36.3) | 0 | 34.0  (17.9 – 66.6) | 0 |  |
| Mono-(2-ethyl-5oxohexyl)phthalate (mEOHP) (nmol/L) | 0.14 | 26.4  (12.1 – 52.3) | 0 | 23.9  (11.8 – 54.0) | 0 | 24.4  (13.2 – 47.4) | 0.1 |  |
| Mono-[(2-carboxymethyl)-hexyl] phthalate (mCMHP) (nmol/L) | 0.13 | 44.7  (24.5 – 82.9) | 0.1 | 12.6  (7.0 – 23.1) | 0.2 | 11.0  (5.8 – 20.6) | 1.1 |  |
| ***Di-n-octylphthalate (DNOP)*** | **-** | 5.7  (3.0 – 10.6) | - | 3.5  (2.0 – 6.6) | - | 7.1  (3.8 – 12.4) | - | 0.22 |
| Mono(3-carboxypropyl)- phthalate (mCPP) (nmol/L) | 0.03 | 5.7  (3.0 – 10.6) | 0 | 3.5  (2.0 – 6.6) | 0 | 7.0  (3.8 – 12.4) | 0.1 |  |
| **Bisphenols** (nmol/L) | **-** | 9.2  (3.5 – 21.0) | - | 6.3  (3.0 – 13.5) | - | 9.1  (4.4 – 18.9) | - | 0.06 |
| Bisphenol A (BPA) (nmol/L) | 0.66 | 4.8  (1.0 – 12.8) | 22.2 | 5.8  (2.6 – 12.4) | 7.3 | 6.5  (2.8 – 13.0) | 9.0 | 0.08 |
| Bisphenol S (BPS) (nmol/L) | 0.20 | 0.7  (<LOD – 2.4) | 31.7 | <LOD  (<LOD - 0.4) | 70.5 | NA | 80.7 | NA |
| Bisphenol F (BPF) (nmol/L) | 0.86 | <LOD  (<LOD – 2.2) | 59.6 | NA | 88.4 | <LOD  (<LOD - 2.4) | 71.5 | NA |

Values represent medians (25^th^-75^th^ percentiles). Absolute urine concentration of the limit of detection (in nmol/L urine), grouped exposures (in nmol/L urine), and individual exposures (in nmol/L urine) with concentrations below the limit of detection imputed as limit of detection/square root of 2. Only values that are included in the calculation of the grouped exposures are included in this table.

ICC: Intraclass Correlation Coefficients calculated using a single measurement, absolute agreement, and two-way mixed-effects model using the non-creatinin corrected (nmol/L) natural log transformed urine concentrations; NA: not applicable due to >80% of concentrations below limit of detection.

**Supplemental Figure 2.** Directed Acyclic Graph showing the hypothesized relationship between fetal exposure to phthalates and bisphenols, childhood blood pressure and the covariates.


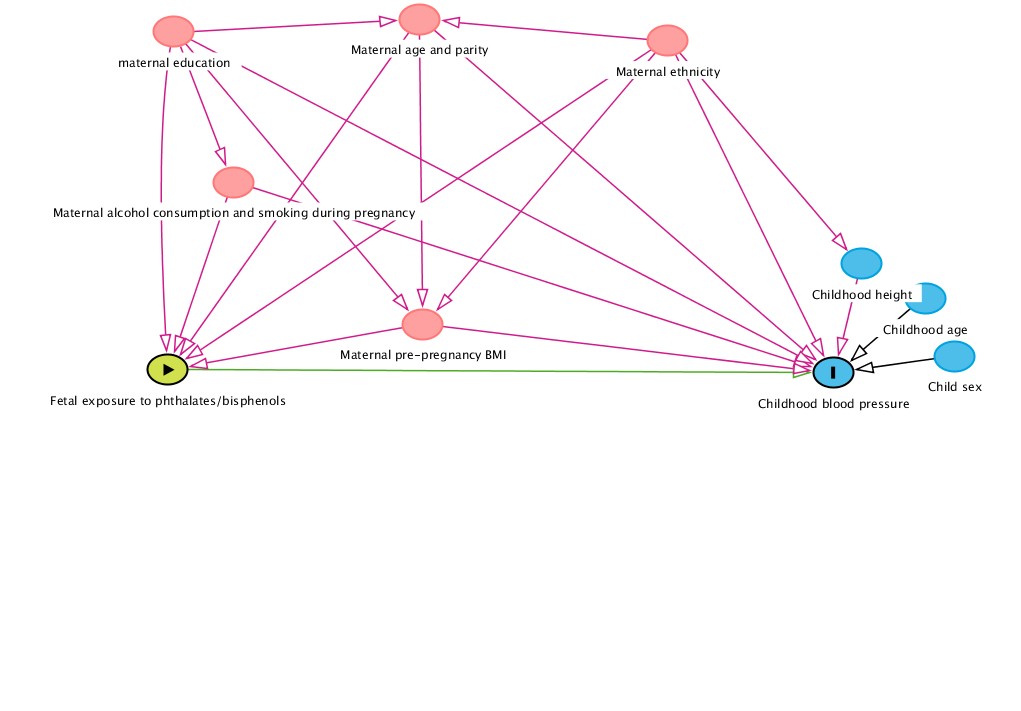


**Supplemental Table S2.** Urine concentrations of phthalates and bisphenols during pregnancy in participants and non-participants (n = 315).

|  | **First trimester**  **Participants** | **First trimester**  **Non-participants** | **p-value^a^** | **Second trimester**  **Participants** | **Second trimester**  **Non-participants** | **p-value^a^** | **Third trimester**  **Participants** | **Third trimester**  **Non-participants** | **p-value^a^** |
| --- | --- | --- | --- | --- | --- | --- | --- | --- | --- |
|  | Median (25^th^-75^th^ percentile) | Median (25^th^-75^th^ percentile) |  | Median (25^th^-75^th^ percentile) | Median (25^th^-75^th^ percentile) |  | Median (25^th^-75^th^ percentile) | Median (25^th^-75^th^ percentile) |  |
| **Phthalic Acid (PA)** (nmol/L) | 343.1  (180.3 – 724.8) | 344.5  (190.8 – 764.2) | 0.66 | 878.5  (349.1 – 1662.6) | 1083.1  (484.7 – 1909.1) | 0.00 | 402.2  (200.4 – 792.3) | 468.5  (213.7 – 877.9) | 0.12 |
| **Low-molecular-weight phthalates (LMWP)**  (nmol/L) | 1092.6  (429.2 – 2903.0) | 1049.6  (434.0 – 3322.4) | 0.53 | 533.6  (224.3 – 1422.3) | 723.4  (318.4 – 1864.7) | 0.00 | 982.0  (388.4 – 2480.7) | 1 352.8  (503.3 – 3165.3) | 0.01 |
| Monomethyl-phthalate (mMP) (nmol/L) | 29.8  (15.1 – 54.9) | 31.9  (15.8 – 55.3) | 0.48 | 18.9  (9.8 – 34.9) | 20.3  (11.7 – 35.0) | 0.20 | 21.6  (10.9 – 44.5) | 25.4  (11.3 – 43.1) | 0.36 |
| Monoethylphthalate (mEP) (nmol/L) | 713.7  (208.1 – 2437.7) | 705.3  (224.7 – 2690.8) | 0.43 | 341.3  (118.7 – 1090.3) | 496.3  (175.4 – 1468.6) | 0.00 | 642.7  (217.5 – 2055.0) | 900.1  (285.0 – 2563.5) | 0.02 |
| Mono-isobutylphthalate (mIBP) (nmol/L) | 95.9  (42.7 – 203.0) | 97.7  (43.7 – 230.4) | 0.40 | 39.5  (19.6 – 77.5) | 45.6  (25.3 – 115.7) | 0.00 | 75.2  (40.3 – 151.9) | 113.69  (54.5 – 214.3) | 0.00 |
| Mono-n-butylphthalate (mBP) (nmol/L) | 72.8  (30.6 – 138.7) | 70.6  (30.2 – 157.9) | 0.75 | 41.8  (23.3 – 81.3) | 59.6  (29.9 – 103.7) | 0.00 | 53.1  (27.4 – 107.1) | 59.7  (30.1 – 123.0) | 0.07 |
| **High-molecular-weight phthalates (HMWP)** (nmol/L) | 214.6  (114.6 – 394.2) | 241.6  (108.3 – 461.5) | 0.14 | 128.0  (70.4 – 236.5) | 167.3  (90.9 – 307.5) | 0.00 | 165.9  (95.5 – 292.9) | 193.1  (91.1 – 333.4) | 0.11 |
| Monobenzylphthalate (mBzBP) (nmol/L) | 22.1  (8.8 – 47.2) | 23.4  (9.3- 51.7) | 0.48 | 19.9  (8.3 – 40.5) | 26.1  (11.1 – 58.5) | 0.00 | 12.0  (4.1 – 24.5) | 13.3  (5.2 – 28.5) | 0.04 |
| Mono-hexylphthalate (mHxP) (nmol/L) | 0.9  (0.3 – 1.9) | 0.9  (0.2 – 2.2) | 0.76 | NA | NA | NA | NA | NA | NA |
| Mono-2-heptylphthalate (mHpP) (nmol/L) | 2.1  (<LOD – 5.3) | 2.4  (<LOD – 6.6) | 0.28 | NA | NA | NA | NA | NA | NA |
| Monocyclohexyl-phthalate (mCHP) (nmol/L) | <LOD  (<LOD – <LOD) | <LOD  (<LOD – <LOD) | 0.13 | NA | NA | NA | NA | NA | NA |
| ***Di-2-ehtylhexylphthalate (DEHP)*** (nmol/L) | 171.4  (89.2 – 313.5) | 187.1  (90.6 – 376.1).80 – 386.62) | 0.11 | 95.4  (50.9 – 178.3) | 120.3  (65.4 – 224.6) | 0.00 | 138.6  (77.5 – 250.2) | 156.6  (77.8 – 279.8) | 0.13 |
| Mono-(2-ethyl-5-carboxy-pentyl)-phthalate (mECPP) (nmol/L) | 52.1  (26.1 – 99.6) | 56.7  (28.3 – 122.3) | 0.07 | 33.1  (17.9 – 61.9) | 40.3  (21.9 – 82.3) | 0.00 | 57.9  (30.7 – 109.2) | 63.1  (31.4 – 116.2) | 0.21 |
| Mono-(2-ethyl-5-hydroxy-hexyl)-phthalate (mEHHP) (nmol/L) | 40.3  (20.1 – 77.0) | 42.7  (19.2 – 84.3) | 0.32 | 18.2  (10.0 – 36.3) | 22.8  (12.1 – 43.8) | 0.00 | 34.0  (17.9 – 66.6) | 39.0  (18.1 – 73.7) | 0.21 |
| Mono-(2-ethyl-5oxohexyl)phthalate (mEOHP) (nmol/L) | 26.4  (12.1 – 52.3) | 28.7  (11.7 – 56.1) | 0.41 | 23.9  (11.8 – 54.0) | 31.1  (14.9 – 64.9) | 0.00 | 24.4  (13.2 – 47.4) | 28.3  (13.3 – 53.8) | 0.09 |
| Mono-[(2-carboxymethyl)-hexyl] phthalate (mCMHP) (nmol/L) | 44.7  (24.5 – 82.9) | 49.2  (26.2 – 97.0) | 0.08 | 12.6  (7.0 – 23.1) | 16.5  (8.6 – 27.6) | 0.00 | 11.0  (5.8 – 20.6) | 12.0  (6.6 – 23.1) | 0.07 |
| ***Di-n-octylphthalate (DNOP)*** | 5.7  (3.0 – 10.6) | 6.6  (3.1 – 12.1) | 0.23 | 3.5  (2.0 – 6.6) | 4.1  (2.5 – 7.3) | 0.01 | 7.1  (3.8 – 12.4) | 7.2  (3.8 – 13.4) | 0.62 |
| Mono(3-carboxypropyl)- phthalate (mCPP) (nmol/L) | 5.7  (3.0 – 10.6) | 6.6  (3.1 – 12.1) | 0.23 | 3.5  (2.0 – 6.6) | 4.1  (2.5 – 7.3) | 0.01 | 7.0  (3.8 – 12.4) | 7.2  (3.8 – 13.4) | 0.62 |
| **Bisphenols** (nmol/L) | 9.2  (3.5 – 21.0) | 9.2  (3.5 – 19.0) | 0.61 | 6.3  (3.0 – 13.5) | 6.5  (3.4 – 14.9) | 0.16 | 9.1  (4.4 – 18.9) | 9.7  (3.9 – 17.9) | 0.78 |
| Bisphenol A (BPA) (nmol/L) | 4.8  (1.0 – 12.8) | 5.0  (1.3 – 11.5) | 0.98 | 5.8  (2.6 – 12.4) | 6.1  (3.1 – 14.6) | 0.13 | 6.5  (2.8 – 13.0) | 6.7  (2.5 – 14.1) | 0.71 |
| Bisphenol S (BPS) (nmol/L) | 0.7  (<LOD – 2.4) | 0.7  (<LOD – 2.5) | 0.68 | <LOD  (<LOD - 0.4) | <LOD  (<LOD – 0.4) | 0.74 | NA | NA | NA |
| Bisphenol F (BPF) (nmol/L) | <LOD  (<LOD – 2.2) | <LOD  (<LOD – 1.7) | 0.44 | NA | NA | NA | <LOD  (<LOD - 2.4) | <LOD  (<LOD – 3.0) | 0.44 |

Values represent medians (25^th^-75^th^ percentiles). Absolute urine concentration of the limit of detection (in nmol/L urine), grouped exposures (in nmol/L urine), and individual exposures (in nmol/L urine) with concentrations below the limit of detection imputed as limit of detection/square root of 2. Only values that are included in the calculation of the grouped exposures are included in this table. The p-value is based on the Mann-Whitney U test.

NA: not applicable due to >80% of concentrations below limit of detection.

**Supplemental Table S3.** Comparison between participants and non-participants.

|  | **Participants** | **Non-Participants** | **P-value** |
| --- | --- | --- | --- |
|  | n = 1,064 | n = 315 |  |
| **Maternal characteristics** |  |  |  |
| Age at enrolment, mean (SD) (years) | 30.9 (4.6) | 29.4 (5.3) | 0.00 |
| Parity, n (%) |  |  | 0.01 |
| Nullipara | 666 (62.9%) | 173 (55.3%) |  |
| Multipara | 392 (37.1%) | 140 (44.7%) |  |
| Ethnicity, n (%) |  |  | 0.00 |
| European | 692 (65.4%) | 154 (50%) |  |
| Non-European | 366 (34.6%) | 154 (50%) |  |
| Education, n (%) |  |  | 0.00 |
| Low | 67 (6.5%) | 35 (12.1%) |  |
| Middle | 407 (39.5%) | 147 (50.9%) |  |
| High | 556 (54.0%) | 107 (37.0%) |  |
| Pre-pregnancy BMI, median (95% range) (kg/m^2^) | 22.7  (18.5 – 34.9) | 22.9 (18.3 – 35.6) | 0.42 |
| Folic acid supplementation, n (%), yes | 708 (83.2%) | 179 (71.9%) | 0.00 |
| Smoking during pregnancy, n (%), yes | 222 (22.9%) | 83 (30.2%) | 0.01 |
| First trimester, n (%), yes | 189 (20.0%) | 73 (27.3%) | 0.01 |
| Second trimester, n (%), yes | 96 (10.3%) | 39 (15.2%) | 0.03 |
| Third trimester, n (%), yes | 97 (10.5%) | 29 (11.6%) | 0.63 |
| Alcohol consumption during pregnancy (any), n (%), yes | 581 (60.4%) | 130 (47.1%) | 0.00 |
| First trimester, n (%), yes | 492 (52.1%) | 111 (40.5%) | 0.00 |
| Second trimester, n (%), yes | 342 (36.7%) | 66 (26.1%) | 0.00 |
| Third trimester, n (%), yes | 361 (39.1%) | 68 (26.9%) | 0.00 |
| **Child characteristics** |  |  |  |
|  |  |  |  |
| Gender (boys), n (%) | 9.7 (0.2) | 158 (50.2%) | 0.90 |

Values represent mean (SD), median (95% range) or number of subjects (valid %).

**Supplemental Table S4.** Associations of maternal urine phthalate concentration during pregnancy with childhood blood pressure at 10 years, basic model, stratified for boys and girls.

|  |  | **Measures of blood pressure at 10 years**  **(in standard deviation scores, 95% confidence interval)** | | | |
| --- | --- | --- | --- | --- | --- |
|  |  | **Mean systolic blood pressure** | | **Mean diastolic blood pressure** | |
| Exposure | **Trimester** | **Boys** | **Girls** | **Boys** | **Girls** |
| PA | First trimester | 0.07  (-0.03; 0.16) | -0.02  (-0.12; 0.08) | 0.03  (-0.07; 0.13) | -0.04  (-0.14; 0.07) |
|  | Second trimester | 0.09  (-0.02; 0.20) | 0.07  (-0.05; 0.19) | 0.04  (-0.08; 0.16) | 0.04  (-0.09; 0.16) |
|  | Third trimester | 0.05  (-0.05; 0.15) | -0.00  (-0.12; 0.11) | -0.04  (-0.15; 0.07) | -0.11  (-0.22; 0.00) |
|  | Overall mean | 0.08  (-0.02; 0.18) | 0.02  (-0.09; 0.13) | -0.01  (-0.12; 0.09) | -0.01  (-0.12; 0.10) |
| LMWP | First trimester | 0.08  (-0.03; 0.18) | 0.04  (-0.08; 0.15) | 0.07  (-0.04; 0.18) | -0.01  (-0.12; 0.11) |
|  | Second trimester | 0.08  (-0.03; 0.19) | 0.15  (0.03; 0.27)* | -0.04  (-0.16; 0.08) | 0.10  (-0.02; 0.21) |
|  | Third trimester | 0.03  (-0.09; 0.14) | 0.01  (-0.11; 0.13) | -0.02  (-0.14; 0.11) | -0.11  (-0.23; 0.01) |
|  | Overall mean | 0.07  (-0.04; 0.18) | 0.07  (-0.05; 0.18) | 0.03  (-0.09; 0.15) | -0.02  (-0.14; 0.10) |
| HMWP | First trimester | 0.06  (-0.03; 0.16) | -0.03  (-0.13; 0.08) | 0.08  (-0.02; 0.18) | -0.10  (-0.20; 0.01) |
|  | Second trimester | 0.06  (-0.04; 0.15) | 0.00  (-0.10; 0.10) | 0.05  (-0.05; 0.15) | -0.08  (-0.17; 0.02) |
|  | Third trimester | -0.03  (-0.13; 0.07) | -0.16  (-0.25; -0.06)† | -0.03  (-0.13; 0.08) | -0.22  (-.031; -0.12)† |
|  | Overall mean | 0.05  (-0.05; 0.15) | -0.08  (-0.18; 0.01) | 0.06  (-0.05; 0.16) | -0.16  (-0.26; -0.07)† |
| DEHP | First trimester | 0.06  (-0.04; 0.15) | -0.03  (-0.13; 0.08) | 0.07  (-0.03; 0.17) | -0.09  (-0.20; 0.01) |
|  | Second trimester | 0.04  (-0.05; 0.14) | -0.01  (-0.11; 0.08) | 0.06  (-0.04; 0.15) | -0.08  (-0.17; 0.02) |
|  | Third trimester | -0.05  (-0.14; 0.05) | -0.14  (-0.23; -0.05)† | -0.04  (-0.14; 0.06) | -0.20  (-0.29; -0.10)† |
|  | Overall mean | 0.03  (-0.07; 0.13) | -0.08  (-0.18; 0.01) | 0.04  (-0.06; 0.14) | -0.15  (-0.25; -0.06)† |
| DNOP | First trimester | 0.04  (-0.05; 0.12) | -0.04  (-0.14; 0.05) | 0.03  (-0.06; 0.12) | -0.07  (-0.17; 0.03) |
|  | Second trimester | 0.04  (-0.06; 0.13) | -0.05  (-0.16; 0.06) | -0.01  (-0.11; 0.09) | -0.03  (-0.14; 0.08) |
|  | Third trimester | 0.02  (-0.08; 0.11) | -0.13  (-0.22; -0.03)* | -0.02  (-0.12; 0.09) | -0.17  (-0.27; -0.07)† |
|  | Overall mean | 0.06  (-0.03; 0.15) | -0.13  (-0.22; -0.03)* | -0.00  (-0.10; 0.10) | -0.15  (-0.25; -0.05)† |

Values are regression coefficients (95% confidence interval) from linear regression models that reflect the difference in blood pressure in SDS for an interquartile range increase in each natural log-transformed phthalate or bisphenol (in µmol/g creatinine) in boys or girls. Model includes child’s age and standardized height.

* p-value<0.05; † Significant after correction for multiple testing (p-value threshold of 0.0098).

DEHP, di-2-ethylhexylphthalate; DNOP, di-n-octylphthalate; HMWP, high molecular weight phthalate; LMWP, low molecular weight phthalate; PA, phthalic acid.

**Supplemental Table S5.** Associations of maternal urine phthalate concentration during pregnancy with childhood blood pressure at 10 years, mutually adjusted model, stratified for boys and girls.

|  |  | **Measures of blood pressure at 10 years**  **(in standard deviation scores, 95% confidence interval)** | | | |
| --- | --- | --- | --- | --- | --- |
|  |  | **Mean systolic blood pressure** | | **Mean diastolic blood pressure** | |
| Exposure | **Trimester** | **Boys** | **Girls** | **Boys** | **Girls** |
| PA | First trimester | 0.04  (-0.06; 0.13) | -0.03  (-0.13; 0.08) | 0.02  (-0.08; 0.12) | -0.02  (-0.12; 0.09) |
|  | Second trimester | 0.05  (-0.06; 0.17) | 0.02  (-0.10; 0.14) | 0.01  (-0.11; 0.14) | 0.02  (-0.11; 0.14) |
|  | Third trimester | 0.02  (-0.08; 0.12) | -0.02  (-0.14; 0.09) | -0.06  (-0.17; 0.05) | -0.12  (-0.23; 0.00)* |
| LMWP | First trimester | 0.03  (-0.08; 0.14) | 0.03  (-0.11; 0.14) | 0.07  (-0.05; 0.19) | 0.01  (-0.12; 0.14) |
|  | Second trimester | 0.05  (-0.07; 0.17) | 0.12  (-0.01; 0.25) | -0.08  (-0.20; 0.05) | 0.12  (-0.01; 0.25) |
|  | Third trimester | -0.02  (-0.14; 0.10) | -0.06  (-0.19; 0.08) | -0.03  (-0.16; 0.10) | -0.16  (-0.29; -0.03)* |
| HMWP | First trimester | 0.03  (-0.07; 0.12) | -0.02  (-0.13; 0.07) | 0.06  (-0.04; 0.16) | -0.06  (-0.17; 0.04) |
|  | Second trimester | 0.05  (-0.04; 0.15) | -0.03  (-0.13; 0.07) | 0.05  (-0.05; 0.15) | -0.08  (-0.18; 0.02) |
|  | Third trimester | -0.04  (-0.14; 0.06) | -0.14  (-0.23; -0.04)† | -0.03  (-0.14; 0.07) | -0.19  (-0.29; -0.10)† |
| DEHP | First trimester | 0.03  (-0.07; 0.12) | -0.02  (-0.13; 0.08) | 0.05  (-0.06; 0.15) | -0.06  (-0.17; 0.04) |
|  | Second trimester | 0.03  (-0.07; 0.13) | -0.03  (-0.13; 0.07) | 0.06  (-0.04; 0.16) | -0.07  (-0.17; 0.03) |
|  | Third trimester | -0.04  (-0.14; 0.06) | -0.12  (-0.21; -0.03)* | -0.04  (-0.15; 0.06) | -0.17  (-0.26; -0.08)† |
| DNOP | First trimester | 0.01  (-0.08; 0.10) | -0.02  (-0.12; 0.08) | 0.02  (-0.07; 0.12) | -0.04  (-0.14; 0.06) |
|  | Second trimester | 0.03  (-0.07; 0.13) | -0.05  (-0.16; 0.06) | -0.02  (-0.12; 0.09) | -0.02  (-0.13; 0.09) |
|  | Third trimester | 0.02  (-0.08; 0.12) | -0.10  (-0.20; -0.00)* | -0.02  (-0.12; 0.09) | -0.16  (-0.26; -0.06)† |

Values are regression coefficients (95% confidence interval) from linear regression models that reflect the difference in blood pressure in SDS for an interquartile range increase in each natural log-transformed phthalate (in µmol/g creatinine) in boys or girls. Model includes child’s age and standardized height and maternal age, education, parity, ethnicity, pre-pregnancy body mass index, alcohol consumption and smoking habits (specifically in early, mid and late pregnancy or during pregnancy).

* p-value<0.05; † Significant after correction for multiple testing (p-value threshold of 0.0098).

DEHP, di-2-ethylhexylphthalate; DNOP, di-n-octylphthalate; HMWP, high molecular weight phthalate; LMWP, low molecular weight phthalate; PA, phthalic acid; SDS, standard deviation score.

**Supplemental Table S6.** Associations of maternal urine phthalate and bisphenol concentration during pregnancy with childhood blood pressure at 10 years, stratified for boys and girls, overall mean.

|  |  | **Measures of blood pressure at 10 years**  **(in standard deviation scores, 95% confidence interval)** | | | |
| --- | --- | --- | --- | --- | --- |
|  |  | **Mean systolic blood pressure** | | **Mean diastolic blood pressure** | |
| Exposure | **Trimester** | **Boys** | **Girls** | **Boys** | **Girls** |
| PA | Overall mean | 0.05  (-0.05; 0.15) | -0.02  (-0.13; 0.09) | -0.04  (-0.15; 0.07) | -0.03  (-0.14; 0.09) |
| LMWP | Overall mean | 0.02  (-0.09; 0.14) | 0.04  (-0.08; 0.16) | -0.01  (-0.13; 0.12) | -0.02  (-0.14; 0.09) |
| HMWP | Overall mean | 0.02  (-0.08; 0.12) | 0.10  (-0.20; -0.00)* | 0.04  (-0.07; 0.14) | -0.17  (-0.26; -0.07)† |
| DEHP | Overall mean | 0.01  (-0.08; 0.11) | -0.09  (-0.19; 0.00) | 0.03  (-0.07; 0.14) | -0.15  (-0.25; -0.06)† |
| DNOP | Overall mean | 0.06  (-0.03; 0.15) | -0.12  (-0.22; -0.02)* | 0.00  (-0.10; 0.10) | -0.15  (-0.24; -0.05)† |
| BP | Overall mean | 0.07  (-0.03; 0.17) | -0.04  (-0.15; 0.06) | 0.06  (-0.05; 0.17) | -0.05  (-0.16; 0.06) |
| BPA | Overall mean | 0.08  (-0.02; 0.17) | -0.07  (-0.17; 0.04) | 0.05  (-0.06; 0.16) | -0.04  (-0.15; 0.06) |
| BPS | Overall mean | NA | NA | NA | NA |
| BPF | Overall mean | NA | NA | NA | NA |

Values are regression coefficients (95% confidence interval) from linear regression models that reflect the difference in blood pressure in SDS for an interquartile range increase in each natural log-transformed phthalate or bisphenol (in µmol/g creatinine) in boys or girls. Model includes child’s age and standardized height and maternal age, education, parity, ethnicity, pre-pregnancy body mass index and alcohol consumption and smoking habits during pregnancy.

* p-value<0.05; † Significant after correction for multiple testing (p-value threshold of 0.0098).

BP, bisphenols; BPA, bisphenol A; BPF, bisphenol F; BPS, bisphenol S; DEHP, di-2-ethylhexylphthalate; DNOP, di-n-octylphthalate; HMWP, high molecular weight phthalate; LMWP, low molecular weight phthalate; NA, not applicable due to >80% of concentrations below limit of detection; PA, phthalic acid; SDS, standard deviation score.

**Supplemental** **Table S7.** Associations of maternal urine individual phthalate concentration during pregnancy with childhood blood pressure at 10 years, stratified for boys and girls.

|  | | **Measures of blood pressure at 10 years**  **(in standard deviation scores, 95% confidence interval)** | | | |
| --- | --- | --- | --- | --- | --- |
|  |  | **Mean systolic blood pressure** | | **Mean diastolic blood pressure** | |
| **Exposure** | **Trimester** | **Boys** | **Girls** | **Boys** | **Girls** |
| **mMP** | First trimester | -0.05  (-0.13; 0.04) | -0.05  (-0.15; 0.04) | -0.04  (-0.13; 0.06) | -0.05  (-0.14; 0.05) |
|  | Second trimester | -0.02  (-0.10; 0.06) | -0.07  (-0.15; 0.01) | -0.03  (-0.12; 0.05) | -0.07  (-0.16; 0.01) |
|  | Third trimester | -0.04  (-0.12; 0.03) | -0.06  (-0.14; 0.03) | -0.08  (-0.16; 0.01) | -0.05  (-0.14; 0.04) |
| **mEP** | First trimester | 0.06  (-0.05; 0.16) | 0.02  (-0.10; 0.15) | 0.06  (-0.06; 0.17) | 0.01  (-0.11; 0.13) |
|  | Second trimester | 0.06  (-0.05; 0.18) | 0.10  (-0.02; 0.22) | -0.04  (-0.16; 0.09) | 0.08  (-0.04; 0.20) |
|  | Third trimester | -0.04  (-0.15; 0.08) | -0.02  (-0.15; 0.10) | -0.06  (-0.19; 0.06) | -0.10  (-0.23; 0.03) |
| **mIBP** | First trimester | 0.06  (-0.03; 0.15) | 0.06  (-0.04; 0.16) | 0.08  (-0.02; 0.18) | -0.01  (-0.11; 0.09) |
|  | Second trimester | 0.04  (-0.06; 0.14) | 0.07  (-0.04; 0.18) | 0.02  (-0.09; 0.13) | -0.03  (-0.14; 0.08) |
|  | Third trimester | 0.09  (-0.01; 0.18) | -0.00  (-0.11; 0.11) | 0.06  (-0.04; 0.16) | -0.13  (-0.24; -0.02) |
| **mBP** | First trimester | 0.05  (-0.03; 0.14) | 0.03  (-0.07; 0.12) | 0.10  (0.10; 0.20)* | 0.01  (-0.09; 0.10) |
|  | Second trimester | 0.05  (-0.05; 0.14) | 0.04  (-0.05; 0.13) | 0.06  (-0.04; 0.15) | -0.00  (-0.10; 0.09) |
|  | Third trimester | 0.07  (-0.03; 0.16) | -0.04  (-0.15; 0.07) | 0.07  (-0.03; 0.18) | -0.12  (-0.23; -0.01)* |
| **mBzBP** | First trimester | 0.08  (-0.02; 0.17) | -0.06  (-0.17; 0.04) | 0.09  (-0.01; 0.19) | -0.06  (-0.17; 0.04) |
|  | Second trimester | 0.02  (-0.07; 0.12) | -0.04  (-0.14; 0.06) | 0.01  (-0.09; 0.12) | -0.13  (-0.23; -0.02)* |
|  | Third trimester | -0.02  (-0.13; 0.09) | -0.20  (-0.31; -0.09)† | 0.04  (-0.07; 0.15) | -0.23  (-0.34; -0.12)† |
| **mHxP** | First trimester | 0.02  (-0.08; 0.11) | -0.02  (-0.14; 0.09) | 0.09  (-0.01; 0.20) | -0.02  (-0.13; 0.10) |
|  | Second trimester | NA | NA | NA | NA |
|  | Third trimester | NA | NA | NA | NA |
| **mHpP** | First trimester | 0.03  (-0.07; 0.14) | -0.02  (-0.13; 0.09) | 0.08  (-0.04; 0.19) | -0.01  (-0.13; 0.10) |
|  | Second trimester | NA | NA | NA | NA |
|  | Third trimester | NA | NA | NA | NA |
| **mCHP** | First trimester | 0.01  (-0.09; 0.10) | -0.02  (-0.13; 0.09) | 0.03  (-0.07; 0.13) | -0.04  (-0.15; 0.07) |
|  | Second trimester | NA | NA | NA | NA |
|  | Third trimester | NA | NA | NA | NA |
| **mECPP** | First trimester | 0.02  (-0.08; 0.11) | -0.06  (-0.17; 0.04) | 0.04  (-0.06; 0.14) | -0.11  (-0.21; 0.00) |
|  | Second trimester | 0.06  (-0.04; 0.15) | -0.07  (-0.17; 0.03) | 0.08  (-0.02; 0.19) | -0.10  (-0.20; 0.00) |
|  | Third trimester | -0.05  (-0.15; 0.05) | -0.09  (-0.19; -0.00)* | -0.06  (-0.17; 0.04) | -0.12  (-0.21; -0.03)* |
| **mEHHP** | First trimester | -0.00  (-0.10; 0.09) | -0.04  (-0.15; 0.07) | 0.03  (-0.07; 0.13) | -0.06  (-0.17; 0.05) |
|  | Second trimester | 0.06  (-0.04; 0.15) | -0.02  (-0.12; 0.09) | 0.08  (-0.02; 0.18) | -0.09  (-0.20; 0.02) |
|  | Third trimester | -0.03  (-0.13; 0.07) | -0.11  (-0.21; -0.01)* | -0.01  (-0.12; 0.09) | -0.20  (-0.30; -0.10)† |
| **mEOHP** | First trimester | 0.03  (-0.07; 0.13) | -0.04  (-0.15; 0.07) | 0.07  (-0.04; 0.17) | -0.06  (-0.17; 0.05) |
|  | Second trimester | 0.03  (-0.07; 0.12) | 0.00  (-0.11; 0.11) | 0.06  (-0.05; 0.16) | -0.09  (-0.20; 0.03) |
|  | Third trimester | -0.00  (-0.10; 0.10) | -0.17  (-0.26; -0.07)† | -0.00  (-0.11; 0.11) | -0.23  (-0.32; -0.13)† |
| **mCMHP** | First trimester | 0.06  (-0.03; 0.14) | -0.02  (-0.11; 0.07) | 0.04  (-0.05; 0.14) | -0.08  (-0.17; 0.02) |
|  | Second trimester | 0.06  (-0.02; 0.15) | -0.04  (-0.13; 0.05) | 0.03  (-0.06; 0.12) | -0.10  (-0.19; -0.01)* |
|  | Third trimester | 0.02  (-0.06; 0.09) | -0.07  (-0.15; 0.02) | 0.01  (-0.08; 0.09) | -0.11  (-0.19; -0.03)* |
| **mCPP** | First trimester | 0.02  (-0.07; 0.11) | -0.04  (-0.14; 0.06) | 0.02  (-0.08; 0.11) | -0.07  (-0.17; 0.03) |
|  | Second trimester | 0.04  (-0.05; 0.13) | -0.06  (-0.17; 0.05) | -0.01  (-0.11; 0.09) | -0.04  (-0.15; 0.07) |
|  | Third trimester | 0.03  (-0.06; 0.13) | -0.11  (-0.21; -0.01)* | -0.01  (-0.11; 0.09) | -0.17  (-0.26; -0.07)† |

Values are regression coefficients (95% confidence interval) from linear regression models that reflect the difference in blood pressure in SDS for an interquartile range increase in each natural log-transformed phthalate (in µmol/g creatinine) in boys or girls. Model includes child’s age and standardized height and maternal age, education, parity, ethnicity, pre-pregnancy body mass index, alcohol consumption and smoking habits (specifically in early, mid and late pregnancy).

* p-value<0.05; † Significant after correction for multiple testing (p-value threshold of 0.0098).

NA, not applicable due to >80% of concentrations below limit of detection.

**Supplemental Table S8.** Associations of maternal urine phthalate concentration during pregnancy with childhood blood pressure at 10 years in children born at term not at low birth weight, stratified for boys and girls (n = 944).

|  |  | **Measures of blood pressure at 10 years**  **(in standard deviation scores, 95% confidence interval)** | | | |
| --- | --- | --- | --- | --- | --- |
|  |  | **Mean systolic blood pressure** | | **Mean diastolic blood pressure** | |
| **Exposure** | **Trimester** | **Boys**  **n=478** | **Girls**  **n=466** | **Boys**  **n=478** | **Girls**  **n=466** |
| **PA** | First trimester | 0.03  (-0.07; 0.12) | -0.06  (-0.16; 0.05) | -0.02  (-0.12; 0.09) | -0.05  (-0.15; 0.06) |
|  | Second trimester | 0.08  (-0.04; 0.19) | 0.00  (-0.13; 0.13) | 0.01  (-0.12; 0.14) | -0.03  (-0.16; 0.11) |
|  | Third trimester | 0.03  (-0.07; 0.13) | -0.02  (-0.14; 0.10) | -0.06  (-0.17; 0.05) | -0.12  (-0.24; -0.00)* |
| **LMWP** | First trimester | 0.02  (-0.09; 0.13) | 0.01  (-0.11; 0.13) | 0.02  (-0.09; 0.14) | -0.02  (-0.14; 0.10) |
|  | Second trimester | 0.04  (-0.07; 0.15) | 0.11  (-0.02; 0.24) | -0.07  (-0.19; 0.05) | 0.07  (-0.06; 0.19) |
|  | Third trimester | -0.03  (-0.14; 0.09) | -0.02  (-0.15; 0.11) | -0.06  (-0.19; 0.07) | -0.14  (-0.27; -0.01)* |
| **HMWP** | First trimester | 0.03  (-0.07; 0.13) | -0.05  (-0.16; 0.07) | 0.03  (-0.08; 0.14) | -0.09  (-0.20; 0.02) |
|  | Second trimester | 0.07  (-0.03; 0.17) | -0.05  (-0.16; 0.06) | 0.05  (-0.06; 0.16) | -0.14  (-0.24; -0.03)* |
|  | Third trimester | -0.03  (-0.13; 0.07) | -0.13  (-0.23; -0.03)* | -0.06  (-0.17; 0.05) | -0.19  (-0.29; -0.09)† |
| **DEHP** | First trimester | 0.03  (-0.07; 0.14) | -0.05  (-0.17; 0.07) | 0.03  (-0.08; 0.14) | -0.05  (-0.17; 0.07) |
|  | Second trimester | 0.04  (-0.06; 0.14) | -0.01  (-0.14; 0.11) | 0.04  (-0.07; 0.15) | -0.13  (-0.26; -0.01)* |
|  | Third trimester | -0.03  (-0.13; 0.08) | -0.15  (-0.25; -0.04)† | -0.05  (-0.16; 0.06) | -0.19  (-0.29; -0.09)† |
| **DNOP** | First trimester | -0.00  (-0.09; 0.09) | -0.06  (-0.16; 0.05) | -0.01  (-0.11; 0.08) | -0.08  (-0.18; 0.02) |
|  | Second trimester | 0.08  (-0.02; 0.17) | -0.06  (-0.17; 0.06) | 0.01  (-0.10; 0.11) | -0.06  (-0.17; 0.06) |
|  | Third trimester | 0.03  (-0.07; 0.13) | -0.10  (-0.21; 0.00) | -0.03  (-0.14; 0.08) | -0.16  (-0.26; -0.05)† |

Values are regression coefficients (95% confidence interval) from linear regression models that reflect the difference in blood pressure in SDS for an interquartile range increase in each natural log-transformed phthalate or bisphenol (in µmol/g creatinine) in boys or girls that were born at term and not small for gestational age. Model includes child’s age and standardized height and maternal age, education, parity, ethnicity, pre-pregnancy body mass index, alcohol consumption and smoking habits (specifically in early, mid and late pregnancy).

* p-value<0.05; † Significant after correction for multiple testing (p-value threshold of 0.0098).

DEHP, di-2-ethylhexylphthalate; DNOP, di-n-octylphthalate; HMWP, high molecular weight phthalate; LMWP, low molecular weight phthalate; PA, phthalic acid.

**Supplemental Table S9.** Associations of maternal urine bisphenol concentration during pregnancy with childhood blood pressure at 10 years, mutually adjusted model, stratified for boys and girls.

|  |  | **Measures of blood pressure at 10 years**  **(in standard deviation scores, 95% confidence interval)** | | | |
| --- | --- | --- | --- | --- | --- |
|  |  | **Mean systolic blood pressure** | | **Mean diastolic blood pressure** | |
| Exposure | **Trimester** | **Boys** | **Girls** | **Boys** | **Girls** |
| BP | First trimester | 0.04  (-0.07; 0.15) | -0.05  (-0.15; 0.06) | 0.08  (-0.04; 0.19) | 0.02  (-0.09; 0.13) |
|  | Second trimester | 0.13  (0.03; 0.23)† | -0.09  (-0.19; 0.01) | 0.04  (-0.06; 0.15) | -0.14  (-0.24; -0.03)† |
|  | Third trimester | 0.01  (-0.09; 0.11) | 0.01  (-0.10; 0.12) | 0.01  (-0.10; 0.12) | -0.08  (-0.19; 0.04) |
| BPA | First trimester | 0.04  (-0.07; 0.15) | -0.06  (-0.17; 0.05) | 0.04  (-0.06; 0.15) | 0.02  (-0.09; 0.13) |
|  | Second trimester | 0.14  (0.04; 0.23)† | -0.09  (-0.18; 0.01) | 0.05  (-0.06; 0.15) | -0.13  (-0.23; -0.03)* |
|  | Third trimester | -0.01  (-0.10; 0.08) | 0.05  (-0.05; 0.15) | 0.02  (-0.09; 0.12) | -0.02  (-0.13; 0.08) |

Values are regression coefficients (95% confidence interval) from linear regression models that reflect the difference in blood pressure in SDS for an interquartile range increase in each natural log-transformed bisphenol (in µmol/g creatinine) in boys or girls. Model includes child’s age and standardized height and maternal age, education, parity, ethnicity, pre-pregnancy body mass index, alcohol consumption and smoking habits (specifically in early, mid and late pregnancy or during pregnancy). This analysis was not performed in BPS and BPF because >80% of values is below the limit of detection for at least one trimester.

* p-value<0.05; † Significant after correction for multiple testing (p-value threshold of 0.0098).

BP, bisphenols; BPA, bisphenol A; BPF, bisphenol F; BPS, bisphenol S; SDS, standard deviation score.

**Supplemental Table S10.** Associations of maternal urine bisphenol concentration during pregnancy with childhood blood pressure at 10 years, basic model, stratified for boys and girls.

|  |  | **Measures of blood pressure at 10 years**  **(in standard deviation scores, 95% confidence interval)** | | | |
| --- | --- | --- | --- | --- | --- |
|  |  | **Mean systolic blood pressure** | | **Mean diastolic blood pressure** | |
| Exposure | **Trimester** | **Boys** | **Girls** | **Boys** | **Girls** |
| BP | First trimester | 0.05  (-0.06; 0.16) | -0.06  (-0.16; 0.05) | 0.09  (-0.03; 0.20) | 0.01  (-0.10; 0.11) |
|  | Second trimester | 0.13  (0.03; 0.23)† | -0.07  (-0.17; 0.04) | 0.04  (-0.07; 0.15) | -0.11  (-0.21; -0.01)* |
|  | Third trimester | -0.00  (-0.10; 0.10) | 0.01  (-0.10; 0.12) | -0.00  (-0.11; 0.11) | -0.07  (-0.18; 0.05) |
|  | Overall mean | 0.08  (-0.02; 0.18) | -0.05  (-0.15; 0.06) | 0.07  (-0.04; 0.18) | -0.05  (-0.16; 0.05) |
| BPA | First trimester | 0.08  (-0.03; 0.19) | -0.06  (-0.17; 0.04) | 0.07  (-0.05; 0.19) | 0.01  (-0.10; 0.11) |
|  | Second trimester | 0.14  (0.05; 0.24)† | -0.07  (-0.17; 0.03) | 0.05  (-0.05; 0.15) | -0.11  (-0.21; -0.01)* |
|  | Third trimester | -0.01  (-0.11; 0.08) | 0.06  (-0.05; 0.16) | 0.01  (-0.09; 0.17) | -0.02  (-0.12; 0.09) |
|  | Overall mean | 0.10  (-0.00; 0.20) | -0.06  (-0.16; 0.05) | 0.10  (-0.02; 0.23) | -0.04  (-0.14; 0.06) |
| BPS | First trimester | 0.06  (-0.06; 0.17) | -0.04  (-0.16; 0.08) | 0.10  (-0.02; 0.23) | 0.02  (-0.10; 0.14) |
|  | Second trimester | -0.08  (-0.18; 0.02) | 0.04  (-0.06; 0.15) | -0.06  (-0.16; 0.04) | 0.01  (-0.09; 0.12) |
|  | Third trimester | NA | NA | NA | NA |
|  | Overall mean | NA | NA | NA | NA |
| BPF | First trimester | 0.02  (-0.09; 0.13) | -0.08  (-0.19; 0.03) | 0.07  (-0.05; 0.19) | -0.05  (-0.16; 0.06) |
|  | Second trimester | NA | NA | NA | NA |
|  | Third trimester | -0.02  (-0.12; 0.09) | -0.13  (-0.25; -0.01)* | -0.07  (-0.18; 0.05) | -0.13  (-0.24; -0.01)* |
|  | Overall mean | NA | NA | NA | NA |

Values are regression coefficients (95% confidence interval) from linear regression models that reflect the difference in blood pressure in SDS for an interquartile range increase in each natural log-transformed phthalate or bisphenol (in µmol/g creatinine) in boys or girls. Model includes child’s age and standardized height.

* p-value<0.05; † Significant after correction for multiple testing (p-value threshold of 0.0098).

BP, bisphenols; BPA, bisphenol A; BPF, bisphenol F; BPS, bisphenol S; NA, not applicable due to >80% of concentrations below limit of detection; SDS, standard deviation score.

**Supplemental Table S11.** Associations of maternal urine bisphenol concentration during pregnancy with childhood blood pressure at 10 years in children born at term not at low birth weight, stratified for boys and girls (n = 944).

|  |  | **Measures of blood pressure at 10 years**  **(in standard deviation scores, 95% confidence interval)** | | | |
| --- | --- | --- | --- | --- | --- |
|  |  | **Mean systolic blood pressure** | | **Mean diastolic blood pressure** | |
| **Exposure** | **Trimester** | **Boys**  **n=478** | **Girls**  **n=466** | **Boys**  **n=478** | **Girls**  **n=466** |
| **BP** | First trimester | 0.04  (-0.07; 0.16) | -0.09  (-0.21; 0.02) | 0.06  (-0.07; 0.18) | -0.01  (-0.12; 0.10) |
|  | Second trimester | 0.14  (0.04; 0.24)† | -0.10  (-0.21; 0.01) | 0.07  (-0.04; 0.18) | -0.12  (-0.23; -0.01)* |
|  | Third trimester | -0.03  (-0.13; 0.08) | 0.02  (-0.10; 0.14) | 0.00  (-0.11; 0.11) | -0.05  (-0.16; 0.07) |
| **BPA** | First trimester | 0.06  (-0.06; 0.18) | -0.11  (-0.22; 0.01) | 0.03  (-0.10; 0.16) | -0.00  (-0.12; 0.11) |
|  | Second trimester | 0.14  (0.05; 0.24)† | -0.10  (-0.21; 0.01) | 0.08  (-0.03; 0.18) | -0.13  (-0.23; -0.02)* |
|  | Third trimester | -0.04  (-0.14; 0.06) | 0.07  (-0.04; 0.18) | 0.02  (-0.09; 0.12) | 0.01  (-0.10; 0.12) |
| **BPS** | First trimester | 0.08  (-0.04; 0.21) | -0.05  (-0.18; 0.08) | 0.10  (-0.03; 0.23) | -0.01  (-0.14; 0.12) |
|  | Second trimester | -0.02  (-0.12; 0.09) | 0.07  (-0.04; 0.18) | -0.03  (-0.14; 0.08) | 0.03  (-0.08; 0.14) |
|  | Third trimester | NA | NA | NA | NA |
| **BPF** | First trimester | 0.04  (-0.08; 0.16) | -0.07  (-0.19; 0.05) | 0.07  (-0.06; 0.19) | -0.03  (-0.16; 0.09) |
|  | Second trimester | NA | NA | NA | NA |
|  | Third trimester | 0.00  (-0.11; 0.11) | -0.12  (-0.25; 0.00) | -0.09  (-0.21; 0.03) | -0.13  (-0.25; -0.00)* |

Values are regression coefficients (95% confidence interval) from linear regression models that reflect the difference in blood pressure in SDS for an interquartile range increase in each natural log-transformed bisphenol (in µmol/g creatinine) in boys or girls born at term and not small for gestational age. Model includes child’s age and standardized height and maternal age, education, parity, ethnicity, pre-pregnancy body mass index, alcohol consumption and smoking habits (specifically in early, mid and late pregnancy).

* p-value<0.05; † Significant after correction for multiple testing (p-value threshold of 0.0098).

BP, bisphenols; BPA, bisphenol A; BPF, bisphenol F; BPS, bisphenol S; NA: not applicable due to >80% of concentrations below limit of detection.
